# Revisiting the relation between syntax, action, and left BA44

**DOI:** 10.3389/fnhum.2022.923022

**Published:** 2022-09-23

**Authors:** David Kemmerer

**Affiliations:** ^1^Department of Speech, Language, and Hearing Sciences, Purdue University, West Lafayette, IND, United States; ^2^Department of Psychological Sciences, Purdue University, West Lafayette, IND, United States

**Keywords:** language, syntax, action, tool use, embodied cognition, mirror neuron areas, Broca’s area, BA44

## Abstract

Among the many lines of research that have been exploring how embodiment contributes to cognition, one focuses on how the neural substrates of language may be shared, or at least closely coupled, with those of action. This paper revisits a particular proposal that has received considerable attention—namely, that the forms of hierarchical sequencing that characterize both linguistic syntax and goal-directed action are underpinned partly by common mechanisms in left Brodmann area (BA) 44, a cortical region that is not only classically regarded as part of Broca’s area, but is also a core component of the human Mirror Neuron System. First, a recent multi-participant, multi-round debate about this proposal is summarized together with some other relevant findings. This review reveals that while the proposal is supported by a variety of theoretical arguments and empirical results, it still faces several challenges. Next, a narrower application of the proposal is discussed, specifically involving the basic word order of subject (S), object (O), and verb (V) in simple transitive clauses. Most languages are either SOV or SVO, and, building on prior work, it is argued that these strong syntactic tendencies derive from how left BA44 represents the sequential-hierarchical structure of goal-directed actions. Finally, with the aim of clarifying what it might mean for syntax and action to have “common” neural mechanisms in left BA44, two different versions of the main proposal are distinguished. Hypothesis 1 states that the very same neural mechanisms in left BA44 subserve some aspects of hierarchical sequencing for syntax and action, whereas Hypothesis 2 states that anatomically distinct but functionally parallel neural mechanisms in left BA44 subserve some aspects of hierarchical sequencing for syntax and action. Although these two hypotheses make different predictions, at this point neither one has significantly more explanatory power than the other, and further research is needed to elaborate and test them.

## Introduction

A substantial amount of research on embodied cognition has focused on action, especially in the context of the Mirror Neuron System (MNS), which consists of bilateral parietofrontal circuits connecting regions of the intraparietal sulcus, supramarginal gyrus, precentral gyrus (including both premotor and primary motor cortices), and posterior inferior frontal gyrus [especially Brodmann area (BA) 44]. The MNS has received tremendous attention for several reasons. Not only does it appear to facilitate the perception and understanding of observed actions by mapping them onto the onlooker’s own motor repertoire (for reviews see Rizzolatti et al., 2014; Urgesi et al., 2014; Bonini, 2017; Hardwick et al., 2018), but its operation is also modulated by myriad factors involving the action, the actor, the observer, the relationship between actor and observer, and the situation (for reviews see Campbell and Cunnington, 2017; Aziz-Zadeh et al., 2018; Amoruso and Finisguerra, 2019; Kemmerer, 2021).

Ever since the MNS was discovered roughly 30 years ago, there has been considerable interest in its relation to language. One line of work has concentrated on the motor features of verb meanings—e.g., the different kinds of movement denoted by *bite, grab*, and *stomp*—with the aim of determining whether these semantic specifications share neural mechanisms with the production and observation of action. Evidence for partial overlap between verb processing and action production, as well as between verb processing and action observation, has been obtained from many experiments using diverse brain mapping methods (for reviews see Kemmerer, 2015a,^2022^; Birba et al., 2017; Cotelli et al., 2018; Pulvermüller, 2018). But several studies have either failed to replicate these results or posed other challenges (Postle et al., 2008; Schuil et al., 2013; Papeo et al., 2015; Vannuscorps et al., 2016; de Zubicaray et al., 2021). Moreover, so far only a few studies have directly compared all three conditions—i.e., verb processing, action production, and action observation—and the outcomes have been inconsistent (Postle et al., 2008; Rueschemeyer et al., 2014; Zhang et al., 2018). Hence, further research is needed to explore these complex functional-anatomical relationships more carefully.

**FIGURE 1:**
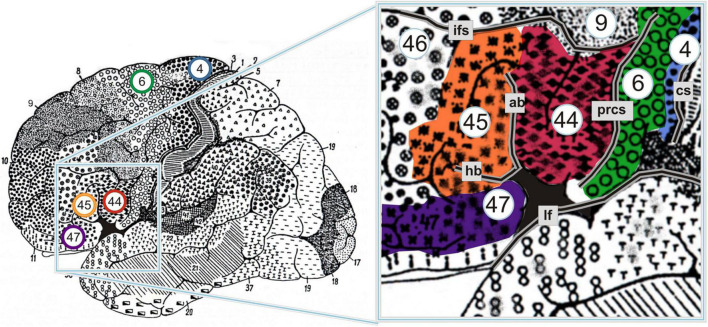
Cytoarchitectonic map of Brodmann areas (BAs) on the lateral surface of the left hemisphere. BAs 44 and 45 are traditionally regarded as comprising Broca’s area; some scholars argue, however, that BA47 should be grouped together with the other two to form “Broca’s complex” (for controversies over the definition of Broca’s area, see Tremblay and Dick, 2016). Note that the deep sulcal portions of these areas are not visible here. ab, ascending branch of the lateral fissure; cs, central sulcus; hb, horizontal branch of the lateral fissure; ifs, inferior frontal sulcus; lf, lateral fissure; prcs, precentral sulcus. From Amunts et al. (2010), p. 2. This material is open access.

Some additional lines of work have been concerned with other ways in which the neural substrates of language may be shared, or at least closely coupled, with those of action, particularly in left BA44—a cortical region that corresponds roughly to the pars opercularis of the inferior frontal gyrus, and that is not only classically regarded as part of Broca’s area, but is also a core component of the MNS (Figure 1). For example, building on two premises—first, that BA44 is the homolog of monkey area F5, and second, that area F5 houses mirror neurons for arm/hand actions—Rizzolatti and Arbib (1998, p. 151) sketched an evolutionary scenario in which those mirror neurons provided “a necessary bridge from ‘doing’ to ‘communicating,’ as the link between actor and observer became a link between the sender and the receiver of each message” (for further elaboration see Arbib, 2005, 2012, Arbib, 2013a,b). Taking a different approach, other researchers have been examining the more specific hypothesis that left BA44 is critically involved in the human propensity to impose sequential and hierarchical structure on multiple domains of mental representation, including not only action and language, but also music, mathematics, and other kinds of auditory and visuospatial stimuli (for reviews see Fiebach and Schubotz, 2006; Tettamanti and Weniger, 2006; Fadiga et al., 2009; Koelsch, 2011, 2012; Uddén and Bahlmann, 2012; Fitch and Martins, 2014; Dehaene et al., 2015; see also Asano et al., 2022).

Picking up on this last theme, the purpose of this paper is to revisit the proposal that left BA44 contains common mechanisms for representing the sequential-hierarchical structure of both linguistic syntax and goal-directed action (see the Appendix for further information about the putative roles of left BA44 in these domains). The first section begins by reviewing a recent multi-participant, multi-round debate about this proposal, and then it summarizes some other recent findings. The upshot is that even though the proposal is supported by a growing body of data, it still faces several challenges. Next, the second section focuses more narrowly on how the proposal applies to the basic word order of subject (S), object (O), and verb (V) in simple transitive clauses. It is well-established that the vast majority of languages are either SOV or SVO, and in previous papers I argued that these strong syntactic tendencies derive from how left BA44 represents the sequential-hierarchical structure of goal-directed actions (Kemmerer, 2012, 2015b). Here, I briefly review this account and expand on it by discussing more recent work. Finally, the third section returns to the general proposal mentioned above and attempts to refine it by outlining two separate versions that—as the text leading up to this last section reveals—have not been clearly distinguished in the literature. According to one version, the hypothesis that left BA44 has “common” mechanisms for representing some aspects of the sequential-hierarchical structure of both linguistic syntax and goal-directed action means that some of the very same neural populations in this region are recruited during the processing of the two domains. According to the other version, the term “common” refers only to functional similarity, with the hypothesis being that left BA44 contains anatomically separate but computationally equivalent mechanisms for the two domains. At this point, both hypotheses are still viable, and further research is needed to formulate them more precisely and determine which has greater explanatory power.

## The controversial relation between syntax, action, and left BA44

### A recent debate

The proposal that left BA44 contains common mechanisms for representing the sequential-hierarchical structure of both linguistic syntax and goal-directed action was the topic of a recent debate that took place in a series of papers published between 2009 and 2015. The main points are reviewed below.

#### Initial argument by Fadiga et al. (2009) and Pulvermüller and Fadiga (2010)

The proposal was initially developed by Fadiga et al. (2009) and Pulvermüller and Fadiga (2010), henceforth referred to as F&PF. Regarding syntax, they cite several fMRI studies that found left BA44 activity in the following contrasts involving receptive sentence processing: syntactically complex vs. simple sentences (e.g., Caplan et al., 2002); syntactically ambiguous vs. unambiguous sentences (e.g., Fiebach et al., 2004); and hierarchical vs. adjacent dependencies (e.g., Bahlmann et al., 2008). Although they could have mentioned extant PET and fMRI evidence that left BA44 also subserves syntactic encoding during sentence production (Indefrey et al., 2001, 2004; Haller et al., 2005), they neglected to do so. On the other hand, they did describe a study which showed that six stroke patients with maximal lesion overlap in left BA44 were impaired at taking scrambled sentences and reordering the words in the correct sequences (Fazio et al., 2009).

Regarding action, F&PF note that left BA44 is engaged during both the execution and observation of goal-directed actions (they cite, among other papers, a review by Binkofski and Buccino, 2006). In addition, they refer to several studies which showed that damage to left BA44 disrupts relevant aspects of non-linguistic action understanding. For instance, two studies linked left BA44 damage with deficits in, first, comparing the attributes of photographed actions (Tranel et al., 2003), and second, matching line drawings of pantomimed actions with the correct objects (Saygin et al., 2004). And another study found that 21 stroke patients with left BA44 damage not only manifested limb apraxia, but were also impaired at judging whether the goals of observed actions were attained (Pazzaglia et al., 2008). Finally, and perhaps most importantly, F&PF emphasize that in the same study mentioned at the end of the previous paragraph, the six patients who were impaired at correctly reordering scrambled sentences also displayed the following dissociation on a task that, on each trial, required them to correctly reorder four snapshots drawn from a previously seen video (Fazio et al., 2009). When the videos portrayed non-goal-directed physical events, like a ball rolling down an inclined plane, the patients’ ability to indicate the right sequence of snapshots was comparable to that of healthy participants; however, when the videos portrayed goal-directed human actions, like a man reaching for and grasping a bottle, the patients’ performance was significantly worse than that of healthy participants. Moreover, a subsequent study showed that a similar dissociation could be temporarily induced in healthy participants by delivering repetitive TMS to left BA44 (Clerget et al., 2009).

Bringing these separate strands of work together, F&PF suggest that, at least in some non-trivial respects, linguistic syntax and goal-directed action may have parallel sequential-hierarchical structures that are represented by common neural mechanisms in left BA44. To illustrate some of the structural similarities between these two domains, Pulvermüller and Fadiga (2010, p. 357) use the following examples: “a center-embedded sentence (*The man {whom the dog chased} ran away*) has the same nested structure as a standard jazz piece (theme {solos} modified theme) and complex everyday action sequences (open door {switch on light} close door). In each case, a superordinate sequence surrounds a nested action or sequence (in the inner parentheses).” Pulvermüller and Fadiga (2010, p. 357) then propose that, given the sorts of findings summarized above implicating left BA44 in the sequential-hierarchical organization of both syntax and action, “the principal underlying brain mechanisms might be the same.”

#### Critiques by Tettamanti and Moro (2012) and Moro (2014a)

1F&PF’s papers spurred a great deal of further discussion of the relation between syntax, action, and left BA44, starting with Tettamanti and Moro (2012) and Moro (2014a). Although Tettamanti and Moro (2012) challenge F&PF in several ways, their primary argument hinges on differences between the perceptibility of hierarchical structure in syntax and action. Focusing first on action, they note that, as in the example of embedding the act of switching on a light between the acts of opening and closing a door, the hierarchical structure of behavioral sequences is visible and hence detectible by the part of the MNS that resides in left BA44. Turning to syntax, however, they maintain that its hierarchical structure cannot be captured by the same neural mechanisms, because much of that information cannot be directly perceived in the linear order of words, but is instead hidden in abstract configurations of symbols for grammatical categories. For example, in the sentence *The agent who called the actress was happy*, the agent is the one who was happy, despite the fact that the sentence contains the sequence *the actress was happy.* Tettamanti and Moro (2012, p. 924) conclude that “since some of the core structural properties of syntax are not directly accessible to hearing and vision, as well as to any other bodily senses, an MNS-based account of the structural properties of syntactic competence, or more radically a unitary MNS theory of the relationship between language and the motor system, is not tenable.”

In a subsequent paper Moro (2014a) went on to level additional arguments against F&PF. For instance, he points out that there does not appear to be an analogue for closed-class morphemes in the domain of action. He also draws attention to the importance of syntactic dependencies in language, as illustrated by the following examples:

(1) Acceptable WH-question formation:
  a. *Bill believes that Susan ate a cricket.*
  b. *What does Bill believe that Susan ate?*
(2) Unacceptable WH-question formation:
  a. *Bill believes the rumor that Susan ate a cricket.*
  b. *What does Bill believe the rumor that Susan ate?

Setting aside various technicalities, what matters here is that according to the version of generative syntactic theory that Moro (2014a) espouses, the difference in acceptability between (1b) and (2b) is due to certain constraints on the kinds of hierarchical phrase structures that are possible in human languages. Based on this assumption, Moro (2014a, p. 110) argues that unless similar constraints, as well as other syntactic principles, can also be identified in the domain of action, ‘‘no parallelism can be inferred.^1^ “

#### Response by Pulvermüller (2014) and counter-response by Moro (2014b)

In a follow-up paper Pulvermüller (2014) defends the view advocated by F&PF. Unfortunately, he does not cite, let alone discuss, Tettamanti and Moro’s (2012) paper, preferring to focus on Moro’s (2014a) critique instead. He emphasizes once again that sequential-hierarchical structure is present not only in linguistic syntax but also in goal-directed action, and he goes on to suggest that recursive embedding may even be applied more often in the latter domain than in the former. To elaborate this point, he develops a detailed example involving the everyday routine of teeth-cleaning, noting along the way that “the rule ‘open X, perform some other action, close X’ is applied recursively to yield increasing levels of ‘self-embedding’ of actions {e.g., [Kim opens the toothpaste tube… (opens her mouth…..closes her mouth)…closes the toothpaste tube]}” (Pulvermüller, 2014, p. 219). Interestingly, in the second part of his paper he concedes that language is special in the ways that Moro (2014a) describes, and in other ways too. But rather than dwelling on such differences, he maintains that much could still be learned by investigating the similarities that do seem to exist between syntax and action, since some of them may reflect shared, or at least closely related, neural mechanisms in left BA44.

In a counter-response Moro (2014b) clarifies what he regards as crucial differences between syntax and action. Beginning with syntax, he uses the sentence *The men who he saw were tall* as an example and makes two points about it: first, the embedded clause *who he saw* can only occur in a certain position; and second, in the main clause *The men…..were tall* a syntactic dependency involving plural number agreement obtains between the noun and the verb. Shifting to action, he takes an example from Pulvermüller (2014)—specifically, [open the door [open the bottle, close the bottle] close the door]—and makes the following remarks: “the action [open the bottle, close the bottle] does not have any special distribution and there is no dependency between [open the door] and [close the door], which is the structure where [open the bottle, close the bottle] is ‘embedded.’ In fact, there is no embedding at all; rather, there is just a sequence” (Moro, 2014b, p. 221). He notes, for instance, that one could easily open a door without closing it afterward, whereas one could not violate the requirement of noun-verb number agreement in *The men….were tall.* Based on such considerations, he concludes that “the idea of a ‘syntax of actions’ remains a metaphor if compared with the syntax of any human language” (Moro, 2014b, p. 221).

Before moving on, it’s worth mentioning that Pulvermüller (2014) could have mounted more resistance to Moro’s (2014a) original critique. For instance, he could have strengthened his position by capitalizing on Pastra and Aloimonos’s (2012) linguistically inspired “generative grammar of action,” in which the elements of goal-directed actions are hierarchically combined into nested temporal sequences of increasing complexity, with distinct roles for actors, tools, and affected objects as well as analogues of syntactic movement. Related theoretical proposals by Jackendoff (2007) and Knott (2012) were also available before Pulvermüller (2014) wrote his paper, but he neglected to use them. Although these approaches may not be able to address all of Moro (2014a,b) concerns [not to mention Tettamanti and Moro’s (2012)], they do illuminate many of the parallels between syntax and action that F&PF and Pulvermüller (2014) are interested in.

#### Further discussion by Boeckx and Fujita (2014) and Leung (2015)

After the exchange between Moro (2014a,b) and Pulvermüller (2014) took place, some new figures entered the debate. Boeckx and Fujita (2014) outline two main arguments against Moro (2014a). First, because generative linguists like him have been known to support partial correspondences between syntax and music (Patel, 2008), they should not dismiss partial correspondences between syntax and action. And second, Darwin’s evolutionary theory of descent with modification predicts that if syntax and action have common roots, they should exhibit both superficial differences and deep similarities. For these reasons, Boeckx and Fujita (2014) advocate further scrutiny of the relation between these two domains (see also the discussion of left BA44 by Boecks et al., 2014 and Asano et al., 2022).

Finally, Leung (2015) weighs in by declaring that the whole debate is on shaky ground. According to him, Pulvermüller’s (2014) argument that syntax and action have shared neural substrates is not very rigorous or convincing, “given the elusive relation between syntax and action which underlies the inquiry” (Leung, 2015, p. 1). Yet he is also unimpressed with Moro (2014a,b) view that the “syntax of action” is just a metaphor, since the esoteric principles and operations posited by generative syntactic theory are likewise just “metaphorical expressions which offer one particular vision to describe the derivation/representation of a sentence” (Leung, 2015, p. 1). Not surprisingly, he thinks much more work is needed to move the debate closer to a resolution. Certainly this is true, but it is not helpful to assert, without justification, that the devices posited by generative syntactic theory are merely “metaphorical expressions,” since they actually reflect genuine attempts to capture the kinds of structures that underlie the grammars of human languages.

### Some other recent findings

In connection with the debate reviewed above, it’s notable that some other recent findings bear on the question of whether linguistic syntax and goal-directed action have common sequential-hierarchical representations subserved by left BA44.

First, in a purely behavioral study Roy et al. (2013) compared the kinematics of three groups of children while they moved a bottle from one location to another: (1) Typically Developing (TD) children; (2) children with Fragile-X Syndrome (FXS), who have delayed but normal syntactic embedding; and (3) children with Specific Language Impairment (SLI), who have both delayed and defective syntactic embedding. Whereas the TD and FXS children performed the action as if its sub-phases had a hierarchical structure akin to syntactic embedding, the SLI children performed the action as if its sub-phases were merely juxtaposed. Although this study did not directly examine the potential role of left BA44 in the movement task, the results still suggest that some aspects of sentences and actions have similar structures underpinned by common neural mechanisms.

Second, in another purely behavioral study Koranda et al. (2020) focused on the following similar phenomena in language production and action production: syntactic priming, which is the tendency to re-use a previously generated syntactic structure; and hysteresis, which is the tendency to re-use a previously generated action program. To investigate whether these phenomena reflect a shared, domain-general planning system, the researchers designed an experiment in which a language task involving two alternative word orders was combined with an action task involving two alternative touch-location orders. In each task, prime trials preceding target trials promoted a particular order. Significant priming effects were found not only within each domain, but also between the two domains, which suggests some degree of cross-talk and provides further evidence for parallels between linguistic syntax and goal-directed action. A caveat, however, is that while the observed priming effects involved sequential order, they did not involve hierarchical structure.

Additional support for such parallels comes from several event-related potential (ERP) studies. In neurolinguistics it is well-established that while an N400 is typically triggered by a violation of semantic content (e.g., *I like my coffee with cream and dog*), a very different biphasic response—specifically, an early left anterior negativity (ELAN) followed by a P600—is typically triggered by a violation of syntactic structure (e.g., *Bill admired Susan’s of picture the park;* for reviews see Kaan, 2007; Swaab et al., 2012; Kemmerer, 2022). What’s striking is that essentially the same ERP effects are also evoked by content violations (N400) and structure violations (ELAN + P600) in visually perceived actions (Sitnikova et al., 2008; Cohn et al., 2014; Maffongelli et al., 2015). For example, when Maffongelli et al. (2015) presented participants with a sequence of static pictures depicting a person making coffee, they observed an N400 when a picture showed the person using cola instead of water (a content violation), and they observed a biphasic ELAN + P600 when the order of two adjacent pictures was reversed (a structure violation). In the current context, the fact that the biphasic ELAN + P600 is evoked by structure violations in both syntax and action bolsters the hypothesis that these two domains have common neural mechanisms. Moreover, the ELAN, which may index the initial detection of a structure violation, most likely stems from the left inferior frontal cortex, though whether it stems specifically from left BA44 is not clear (Friederici et al., 1999; Jakuszeit et al., 2013).

Shifting to functional neuroimaging studies, Papitto et al. (2020) recently reported a meta-analysis of 416 PET and fMRI experiments in order to elucidate the degree to which Broca’s area contributes to six kinds of action processing—namely, execution, observation, imitation, imagination, preparation, and learning. They also compared their results with those of Clos et al.’s (2013) meta-analytically based parcellation of BA44 into five distinct clusters, each of which is associated with certain functions, such as particular types of action, language, memory, and cognition. With specific regard to left BA44, Papitto et al. (2020) found overlapping engagement for only three kinds of action processing—namely, execution, imitation, and imagination. This is surprising, however, because evidence that left BA44 also contributes to action observation, and is hence part of the MNS, comes from several sources, including previous meta-analyses of PET and fMRI studies (Caspers et al., 2010; Molenberghs et al., 2012; Hardwick et al., 2018), a synthesis of lesion-symptom mapping studies (Urgesi et al., 2014), and a review of TMS studies (Avenanti et al., 2013). Papitto et al. (2020, p. 10) also state that “direct comparison between the present meta-analytical data for action and those of an earlier work on language (Clos et al., 2013) reveals a non-overlap of the crucial activation in BA44, with the activation pattern for language being more anterior, and the activation pattern for motor processing being more posterior.” Once again, however, this is problematic. Papitto et al. (2020) only compared their results with Clos et al.’s (2013) relatively anterior cluster 3 (C3), and ignored the more posterior cluster 1 (C1). But according to Clos et al. (2013, p. 182), C1 is not only associated with “action imagination and execution as well as with action and body-related perception,” but is also associated with “phonology, syntax, and tasks requiring overt speech.” Thus, contrary to their own conclusion, Papitto et al.’s (2020) findings do not really threaten the hypothesis that syntax and action have common sequential-hierarchical representations in left BA44, and what’s more, Clos et al.’s (2013) findings clearly support this proposal. At the same time, however, it’s important to keep in mind the limited spatial resolution of fMRI. Despite the fact that Clos et al. (2013) found similar responses to syntax and action in C1 of left BA44, the relevant neural mechanisms for these two domains may still be anatomically separate, residing at a spatial scale that is too small for fMRI to detect (This possibility is discussed further in the section called “Refining the hypothesis space.”).

Finally, a number of recent studies have sought to determine whether linguistic syntax and tool use involve, to some extent, parallel sequential-hierarchical representations that rely on left BA44. Two purely behavioral studies provide some support for shared representations. First, Brozzoli et al. (2019) found that participants’ motor proficiency in a task requiring tool use lawfully predicted their syntactic proficiency in a task requiring sentence production. And second, Thibault et al. (2021) found that tool-use training improved syntactic processing and, reciprocally, syntactic training improved tool use. However, fMRI studies have yielded inconsistent results. On the one hand, many studies have found that, like syntax, tool use engages left BA44 (for a review see Lewis, 2006), and one investigation even revealed overlapping activation in left BA44 when Japanese participants listened to a story and when they used chopsticks (Higuchi et al., 2009). On the other hand, when Thibault et al. (2021) employed multivariate pattern analysis to compare participants’ neural responses during two tasks—one involving comprehension of sentences with relative clauses, and the other involving complex tool use—they found similar activation patterns in parts of the basal ganglia but not in left BA44. In particular, within the left inferior frontal gyrus, the syntax task recruited an area more anterior to the one recruited by the tool task. This outcome therefore challenges the hypothesis that syntax and action have shared neural mechanisms in left BA44.

### Summary

A great deal of controversy has surrounded the provocative proposal that some aspects of linguistic syntax and goal-directed action rely, to a non-trivial degree, on common sequential-hierarchical representations that are neurally subserved by left BA44, a brain region that is widely regarded as a key component of the MNS.

On the one hand, this proposal has received support from many empirical discoveries and theoretical ideas. PET and fMRI studies have revealed convergent engagement of left BA44 during the processing of both sentences and actions (e.g., Higuchi et al., 2009; Clos et al., 2013). ERP studies have shown that a biphasic ELAN + P600 is evoked by perceived violations of sequential-hierarchical structure in both sentences and actions, with the ELAN most likely originating from the left inferior frontal cortex, which contains BA44 (Sitnikova et al., 2008; Cohn et al., 2014; Maffongelli et al., 2015). Lesion studies and TMS studies have demonstrated that either permanently damaging or temporarily perturbing left BA44 disrupts the sequential-hierarchical processing of both sentences and actions (Clerget et al., 2009; Fazio et al., 2009). Even though purely behavioral studies cannot disclose the workings of any particular brain region, they have nonetheless uncovered close cognitive correspondences between syntax and action (Roy et al., 2013; Brozzoli et al., 2019; Koranda et al., 2020). Furthermore, computational analyses have shown how such parallels can be explicitly modeled (Jackendoff, 2007; Knott, 2012; Pastra and Aloimonos, 2012).

On the other hand, the proposal has been challenged by several other observations. One concern is that while the hierarchical organization of goal-directed actions is often apparent, that of sentences cannot be directly perceived (Tettamanti and Moro, 2012). In addition, the domain of action not only has no analogue for closed-class items, but also seems to lack some of the idiosyncratic, structure-dependent constraints that apply to syntax Moro (2014a,b). And with respect to experimental studies, a recent fMRI investigation that used multivariate pattern analysis failed to find comparable neural responses in left BA44 during tasks involving syntax and action (Thibault et al., 2021).

Given these discrepant sets of considerations, it is clear that much more research is needed to understand the complex relation between syntax, action, and left BA44.

## The specific case of basic word order

### The crosslinguistic preference for SOV and SVO word orders

One way in which progress could potentially be made would be to focus on one of the simplest and most well-studied manifestations of syntax—specifically, the basic word order of transitive clauses (Kemmerer, 2012, 2015b). In linguistic typology, basic word order is defined as the sequence of subject (S), object (O), and verb (V) that satisfies the following criteria: it is used most frequently; it has the least amount of function-indicating phonological, morphological, or syntactic marking; and it carries no special pragmatic information apart from declarative mood (Dryer, 2007, 2013). Although most languages have a basic word order, some do not, preferring flexibility instead. Among those languages that do have a basic word order, all six of the possible linearizations of S, O, and V are attested. However, ever since Greenberg’s (1963) seminal research on this topic, it has been known that in the vast majority of languages the basic word order is either SOV or SVO, with the former being somewhat more prevalent than the latter. In the most comprehensive survey to date, Dryer (2013) analyzed a global sample of 1,376 spoken languages and found that 1,187 (87%) have a basic word order. Moreover, of those 1,187 languages, 1,052 (89%) are either SOV or SVO, with the former being a bit more common (564, 54%) than the latter (488, 46%).

### A cognitive account in terms of the sequential-hierarchical structure of the prototypical transitive action scenario

Why are most languages either SOV or SVO? Different explanations have been offered, but they all draw upon the notion of “subject salience” (Greenberg, 1963; Tomlin, 1986; Comrie, 1989; Song, 1991; see also Bornkessel et al., 2006; Wilson et al., 2022). One way in which this notion can be elaborated is as follows. Although transitive clauses can describe a tremendous variety of situations, it is widely assumed that they apply fundamentally to events that fit the so-called prototypical transitive action scenario, in which an animate agent performs an action that causes an inanimate patient to undergo a change of state, as in the sentence *Jill cut the ribbon* (Hopper and Thompson, 1980; Croft, 1990; Langacker, 1991; Shibatani, 2006; Naess, 2007). In this scenario the agent is more prominent than the patient, not only because it is animate, but also, and more importantly, because it is the starting point, the instigator, of the event. And crucially, this temporal precedence of the agent’s volitional action over the patient’s resultant transformation is captured, in an iconic or isomorphic manner, by the temporal precedence of the S over the O in transitive clauses that have either SOV or SVO word order. It’s also notable that the prototypical transitive action scenario has a hierarchical structure, since the agent’s behavior is guided by an overarching goal, and this is reflected linguistically by the fact that the words in a transitive clause make up a higher-level syntactic unit.^2^

Still, there remains the question of whether the order of V and O matters. Recent research suggests that this is influenced by whether the entity encoded by O is animate or inanimate. As noted above, in the prototypical transitive action scenario, the agent, which is encoded by S, is animate, whereas the patient, which is encoded by O, is inanimate. Hence, the event is semantically non-reversible, since it can only involve the agent acting on the patient, and not the opposite. Several experiments have shown that when speakers of SOV languages as well as speakers of SVO languages are asked to manually pantomime but not verbally describe events, they tend to produce an agent-patient-action sequence, akin to SOV word order, when the patient is inanimate and the event is semantically non-reversible, as in the prototypical transitive action scenario; however, they tend to produce an agent-action-patient sequence, akin to SVO word order, when the patient is animate and the event is semantically reversible (Gibson et al., 2013; Hall et al., 2013, 2014 see also So et al., 2005; Goldin-Meadow et al., 2008; Langus and Nespor, 2010; Schouwstra, 2012). Moreover, a survey of 42 sign languages revealed that, as in spoken languages, SOV and SVO are the most common patterns; however, in keeping with the pantomime data just mentioned, SOV is favored for non-reversible events and SVO is favored for reversible ones (Napoli and Sutton-Spence, 2014).

These findings suggest that SOV may be the default cognitive strategy for describing events that conform to the prototypical transitive action scenario. This view is consistent with arguments that the earliest human languages were SOV (Givón, 1979; Newmeyer, 2000; Gell-Man and Ruhlen, 2011) and also with the closely related discovery that during a single generation the incipient Al-Sayyid Bedouin Sign Language developed a syntactic structure characterized by SOV order (Sandler et al., 2005). To explain not only the roughly equal proportions of SOV and SVO languages around the world today, but also the apparent preference for SVO to describe reversible events, Gibson et al. (2013) adopt a historical perspective that takes into account the communicative need to maximize information and minimize noise. They argue that if one assumes an original inclination toward SOV, there appear to be two main ways to reduce agent/patient ambiguity when describing reversible events: one involves keeping SOV as the basic word order but adding a case-marking system; and the other involves shifting to SVO as the basic word order, usually without adding a case-marking system. Gibson et al. (2013) provide some support for these conjectures but acknowledge that they require further refinement and testing.

### The involvement of left BA44

In my previous papers on this topic, I suggested that the aforementioned aspects of basic word order may derive from the action-related functions of left BA44. More specifically, I argued that the pivotal role that left BA44 plays in representing the sequential-hierarchical structure of both executed and observed goal-directed actions provided the neural platform for representing, in a more schematic and long-term form, the sequential-hierarchical structure of the prototypical transitive action scenario, which in turn gave rise to the strong crosslinguistic tendency for transitive clauses to be either SOV or SVO (Kemmerer, 2012, 2015b). My aim here is to expand on those papers by highlighting some new developments and pointing out some issues that require further research.

Due to the “subject salience” principle, which reflects the temporal and causal precedence of the agent over the patient in the prototypical transitive action scenario, clauses in which S precedes O should be more computationally tractable for left BA44 than clauses in which O precedes S. In my previous papers I cited several PET and fMRI studies that confirm this prediction, since they found greater left BA44 responses to O-initial than S-initial constructions. More recently, similar results emerged from a meta-analysis of 22 experiments that involved diverse languages (English, German, Hebrew, and Japanese; Meyer and Friederici, 2016). In addition, a TMS study showed that left BA44 is not merely engaged more by O-initial than S-initial constructions, but causally contributes to the greater processing required by the former than the latter (Kuhnke et al., 2017). And another relevant finding was obtained by Meltzer-Asscher et al. (2015), who conducted an fMRI study that revealed stronger left BA44 responses to intransitive verbs like *fall* (which has a non-canonical syntactic-semantic mapping since the S encodes a patient instead of an agent) than to both intransitive verbs like *swim* (which has an agent S) and transitive verbs like *build* (which has an agent S and a patient O).

Turning to a different development, recent fMRI work suggests that during the comprehension of transitive clauses, left BA44 is modulated not only by the order of S and O, but also by whether the designated event involves a physical action or some other state of affairs. The available data, however, are mixed, and their interpretation is not clear. On the one hand, in two separate studies Tettamanti et al. (2005, 2008) found that left BA44 is engaged more by transitive clauses that refer to physical actions than by those that refer to abstract situations, and in another study Baumgaertner et al. (2007) found overlapping left BA44 responses when the following contrasts were conjoined: transitive action sentences vs. transitive non-action sentences, and transitive action videos vs. transitive non-action videos. On the other hand, as Van Dam and Desai (2016) note, in two separate studies Desai et al. (2009, 2011) failed to find greater left BA44 responses to transitive action sentences than to transitive non-action sentences, not only when just the V differed between the two conditions (e.g., *I throw the ball* vs. *I see the ball*), but also when both the V and the O differed (e.g., *I throw the ball* vs. *I take the risk*). Van Dam and Desai (2016) raise the possibility that in those studies that did find stronger left BA44 recruitment for action than non-action sentences, the former may have been longer than the latter, leading to greater speech-related neural activity. But while Tettamanti et al. (2005) did not report the length of their sentence conditions, Tettamanti et al. (2008) matched the length of theirs, and Baumgaertner et al. (2007) indicate that their non-action sentences were actually longer than their action sentences (average auditory duration of 1.9 vs. 1.8 s). Thus, some other factor(s) may be responsible for the inconsistent results across these studies.^3^

A closely related issue that also deserves more attention involves the proper predictions regarding left BA44. Given that this region has been implicated in representing the sequential-hierarchical structure of goal-directed actions, the most natural prediction seems to be that it should respond more strongly to sentences that describe such actions than to sentences that describe other states of affairs, as Tettamanti et al. (2005, 2008) and Baumgaertner et al. (2007) observed. However, one might also expect the opposite response profile for the following reason: non-action sentences might be harder for left BA44 to process than action sentences, leading to greater neural engagement, just as O-initial order is typically harder for left BA44 to process than S-initial order, leading to greater neural engagement. Then again, it may be the case that left BA44 is more sensitive to the linearization of transitive clauses than to the details of their semantic content. Further research is needed to explore these topics.

Another development relates to the question of how left BA44 processes transitive clauses in those rare languages for which the basic word order involves placing O before S. Almost nothing is known about how the mind/brain handles such languages, but a few hints come from recent work on Kaqchikel, an endangered Mayan language spoken in Guatemala (Koizumi et al., 2014, 2020; Yasunaga et al., 2015; Koizumi and Kim, 2016). First, however, the following caveat should be noted. According to Koizumi and Kim (2016, pp. 2 and 6), the word order in Kaqchikel is relatively flexible, and to the extent that it has a basic word order, the criteria of syntactic marking and discourse frequency yield different results: “Although its syntactically basic word order is VOS, SVO is more frequently used,” especially “when the subject is a topic.” Despite these discrepancies, Koizumi and colleagues assume that the basic word order is VOS, and this view is supported by several experimental results. Koizumi et al. (2014) found that VOS sentences are processed faster than SVO and VSO sentences. In addition, Yasunaga et al. (2015) found that, compared to VOS sentences, SVO and VSO sentences trigger a P600, though they did not report whether this effect is preceded by the type of ELAN that has been linked with left BA44. Furthermore, Koizumi and Kim (2016) conducted an fMRI study which revealed that SVO sentences elicit greater activation than VOS sentences, but in left BA47 rather than left BA44 (Figure 1). The researchers do not attempt to explain why this is the case; however, given that left BA47 is associated much more with semantic than syntactic processing (Vigneau et al., 2006), its stronger response to SVO than VOS sentences may reflect semantic rather than syntactic factors. In this connection, it may be relevant that Koizumi et al. (2020, p. 137) recently obtained behavioral evidence that during sentence production the agent is “conceptually more salient than other elements even for Kaqchikel speakers.” Overall, though, it is apparent that further work is needed to explore how left BA44, and other sectors of the left inferior frontal gyrus, contribute not only to syntax but also to action in Kaqchikel as well as other languages for which the basic word order involves placing O before S.

## Refining the hypothesis space

### An evolutionary perspective

As mentioned in the Introduction, humans have a propensity to impose nested tree structures—which is to say, hierarchical patterns—on strings of data in multiple domains of experience, including not only language and action, but also music, mathematics, and other kinds of auditory and visuospatial stimuli. Fitch (2014) uses the novel term “dendrophilia” to characterize our inordinate fondness for hierarchical sequencing, and he argues that it is a uniquely human trait that plays a major role in distinguishing our cognition from that of other species. Several researchers have suggested that our exceptional capacity for sequential-hierarchical representation is underpinned substantially, but by no means entirely, by left BA44 (for reviews see Fiebach and Schubotz, 2006; Tettamanti and Weniger, 2006; Fadiga et al., 2009; Koelsch, 2011, 2012; Uddén and Bahlmann, 2012; Fitch and Martins, 2014; Dehaene et al., 2015). As we have seen, this view is supported by numerous investigations of how left BA44 functions within the human connectome, and other relevant findings come from anatomical comparisons between this region in humans and the homologous region in great apes. For instance, left BA44 is 6.6 times bigger in humans than in chimpanzees, which is a disproportionate difference, since the whole frontal cortex is only 4.6 times bigger, and the entire brain is just 3.6 times bigger (Schenker et al., 2010; see also Smaers et al., 2017; Donahue et al., 2018). In a similar vein but at a microscopic scale, relative to all great ape species, humans have significantly more neuropil volume in left BA44, allowing more space for local and inter-regional connectivity (Palomero-Gallagher and Zilles, 2019; see also Changeux et al., 2021).

These phylogenetic discoveries provide a useful perspective for thinking about how left BA44 evolved to promote the distinctively human predilection for hierarchical sequencing, particularly in the two domains that are the focus of this paper—namely, linguistic syntax and goal-directed action—but in other domains too. With specific regard to syntax and action, we have concentrated on the proposal that some of their sequential-hierarchical structures are subserved by common neural mechanisms in left BA44. However, as mentioned in the Introduction, and as the previous two sections reveal, the literature on this topic is not always clear about what “common” really means in this context. With the aim of helping to move this field forward, two separate hypotheses are outlined below.

### Hypothesis 1: The same neural mechanisms in left BA44 subserve the sequential-hierarchical processing of syntax and action (as well as other domains)

Building on Lashley’s (1951) seminal ideas, Fitch and Martins (2014) flesh out Hypothesis 1 as follows: Left BA44 computes abstract, nested tree structures that are held in working memory as they unfold over time, with higher-level elements being maintained while lower-level ones gradually become active. Crucially, for many of these tree structures, the configuration of nodes is domain-independent, and the nodes themselves are like slots or variables that, during particular instances of online processing, are transiently bound with domain-specific fillers (e.g., sequences of lexical items or sequences of motor representations) that are retrieved from other brain regions (for some hints about the cellular neurophysiology of such variable-filler bindings, see Xie et al., 2022). In other words, while the skeletal geometry of many hierarchical-sequential structures is the same for different domains, the associated content changes depending on which domain is being processed. According to Fitch and Martins (2014, p. 96), left BA44 implements “many types of templates, independent of format (e.g., verbal, melodic, motor), and thus [is] available for multiple domains.” It operates like “a storage buffer scannable by other cortical and subcortical circuits subserving sequential behavior. This buffer is required to implement hierarchical sequence processing, and its processing load increases with the depth and complexity of the hierarchy being processed.”

This formulation of Hypothesis 1 may be a more precise way of capturing (Pulvermüller and Fadiga, 2010, p.357) view that, with respect to the sequential-hierarchical organization of both syntax and action, “the principal underlying brain mechanisms might be the same.” Evidence for Hypothesis 1 comes from several sources, but especially from fMRI, lesion, and TMS studies that have found the same part(s) of left BA44 to be recruited during both syntactic and action processing tasks (Baumgaertner et al., 2007; Clerget et al., 2009; Fazio et al., 2009; Higuchi et al., 2009; Clos et al., 2013). On the other hand, Hypothesis 1 is inconsistent with Thibault et al.’s (2021) recent fMRI study which failed to find overlapping multivariate response patterns in left BA44 during both syntactic and action processing tasks. It is also challenged by Moro (2014a,b) concerns about structural differences between syntax and action; however, as already noted, Boeckx and Fujita (2014) point out that these concerns may be overstated, since Darwin’s evolutionary theory of descent with modification predicts that if syntax and action have common roots, they should exhibit both superficial differences and deep similarities.

### Hypothesis 2: Anatomically distinct but functionally similar neural mechanisms in left BA44 subserve the sequential-hierarchical processing of syntax and action (as well as other domains)

An alternative approach is elaborated by Dehaene et al. (2015, p. 13) as follows: “…in humans, tree structures are ubiquitous: the human brain may exhibit a specific ‘dendrophilia’ (Fitch, 2014) (i.e., a propensity to impose tree structures on virtually any domain of perception, action, or thought). One may formulate the tentative hypothesis that multiple parallel IFG areas…may be involved in the construction of tree structures in different domains.” Thus, Hypothesis 2 holds that left BA44 contains many anatomically distinct yet functionally similar sets of neural mechanisms for hierarchical sequencing, with each set being devoted to a different representational domain, such as syntax and action, and having distinct long-distance connections with other regions in the relevant widely distributed network, such as the language system or the motor system.

This view is supported by Thibault et al.’s (2021) discovery that syntactic and action processing tasks elicit distinct inferior frontal multivariate response patterns. In addition, Hypothesis 2 is better suited than Hypothesis 1 to accommodate not only the similarities but also the differences between the sequential-hierarchical structures of syntax and action. Moreover, because the separate sets of neural mechanisms that Hypothesis 2 posits may have very close proximity to each other, they may not be distinguishable by conventional univariate PET and fMRI methods, let alone TMS and lesion-symptom mapping methods. Hence, the findings that appear to favor Hypothesis 1 may actually be compatible with Hypothesis 2 as well. Much more research is obviously needed, however, to elaborate both hypotheses in greater detail and determine which has more explanatory power.

## Conclusion

A considerable amount of theoretical and empirical work has recently focused on the complex relation between linguistic syntax, goal-directed action, and left BA44. As this review has shown, a number of intriguing ideas about these functional-anatomical links have been developed, and new insights have come from many studies employing diverse brain mapping methods. However, this topic remains contentious, and further research is needed to not only address the many unresolved issues described here, but also elucidate how both the similarities and the differences between syntax and action extend beyond left BA44 to many other cortical and subcortical structures.

## Author contributions

The author confirms being the sole contributor of this work and has approved it for publication.

## Footnotes

1 Other theories suggest that the data in (1) and (2) do not necessarily reflect constraints on phrase structure geometries, but can be explained instead in terms of pragmatic notions like presupposition and focus (Van Valin, 1998). The overarching point of Moro’s critique, however, is that regardless of how the data are explained, it is difficult to find parallel phenomena in the domain of action.

2 Another principle called “verb-object contiguity” maintains that V and O tend to be adjacent because this reflects the tight bond between the agent’s action and its effect on the patient (for details see Kemmerer, 2012). As Arbib (2015) points out, however, this principle does not add any explanatory power beyond “subject salience,” because if S is the first element in a transitive clause, V and O must occur afterward and hence be adjacent. It’s also notable that “subject salience” seems to carry more weight than “verb-object contiguity,” since the third most common ordering pattern, VSO, preserves the former principle at the cost of violating the latter (Song, 1991).

3 In response to a reviewer’s comment, it’s worth noting that mixed results have also been obtained by fMRI studies that explored how left BA44 responds during the processing of transitive clauses that not only have non-basic O-initial word order but also vary in semantic reversibility. Consistent with the general trend mentioned above (see again the meta-analysis by Meyer and Friederici, 2016), Grewe et al. (2007) found greater left BA44 engagement for German O-initial than S-initial constructions, regardless of whether they were semantically reversible. However, whereas Caplan et al. (2008) likewise found greater left BA44 engagement for English O-initial than S-initial constructions when both types were semantically reversible, they did not find such differential engagement when both types were semantically non-reversible. Such mixed results highlight the need for further research to investigate how left BA44 is modulated by various combinations of the following factors: basic vs. non-basic word order; concrete action content vs. abstract non-action content; and semantically reversible vs. non-reversible actions. It’s worth emphasizing that my own hypothesis about the relation between syntax, action, and left BA44 focuses rather narrowly on how clauses with basic word order encode the prototypical transitive action scenario, which is concrete and semantically non-reversible.
