# Supplementary material for: Revisiting the relation between syntax, action, and left BA44
Source: Front Hum Neurosci. 2022 Sep 23;16:923022. doi: 10.3389/fnhum.2022.923022 (PMC9537576; doi:10.3389/fnhum.2022.923022)
Supplement: Supplementary file 1 [file Presentation_1.pdf]

Appendix: The roles of left BA44 in current models of the widely distributed neural networks underlying linguistic syntax and goal-directed action

Sentence processing is subserved by a large-scale network of brain structures, but contemporary models differ in the roles they ascribe to the left inferior frontal gyrus (IFG), and to BA44 in particular (Bornkessel-Schlesewsky & Schlesewsky, 2016, 2019; Friederici, 2016, 2017; Hagoort, 2014, 2016; Kemmerer, 2022; Matchin & Hickok, 2020; Murphy, 2021). For instance, Friederici's (2016, 2017) model focuses on sentence comprehension and postulates that BA44 has the following functions: it supports simple syntactic computations, such as building local phrases, through its interactions with the frontal operculum and anterior superior temporal gyrus (STG); and it supports complex syntactic computations, such as building hierarchical configurations of nonadjacent elements, through its interactions with the posterior STG. Bornkessel-Schlesewsky and Schlesewsky's (2016, 2019) model also focuses on sentence comprehension and emphasizes the dorsal pathway that connects BA44 with the posterior STG; however, it postulates that this pathway is not specialized for complex syntactic computations per se, but is instead critical for the more basic function of sentence-level sequential processing (i.e., tracking word order). Hagoort's (2014, 2016) model applies to both sentence comprehension and sentence production, and it postulates that both BA44 and BA45 subserve syntactic unification, which involves assembling the sequential-hierarchical structures of phrases, clauses, and complex sentences based on the syntactic properties of lexical items stored in the lateral temporal cortex. Finally, Matchin and Hickok's (2020) model postulates that BA44 does not contribute directly to syntax; instead, BA45 supports the sequential processing of sentences during production but not comprehension, via connections with lexical representations in the lateral temporal cortex.

Turning to the domain of action, it is widely agreed that left BA44 is a major node in the parietofrontal circuitry that constitutes the Mirror Neuron System (MNS), but opinions differ regarding the role that it plays. The dominant view is that left BA44 contributes to representing the intentions of goal-directed actions and therefore occupies a relatively high level in the motor hierarchy, facilitating the selection and sequencing of both executed and observed movements. Evidence for this view comes from several studies cited in the main text (Baumbaertner et al., 2007; Clerget et al., 2009; Fazio et al., 2009; Pazzaglia et al., 2008) and from many others as well (see the review by Rizzolatti et al., 2014). On the other hand, an alternative model holds that there is a gradient of action representation along the anterior-posterior axis of the left IFG, with increasingly abstract properties like goal and intention being encoded by more anterior sectors (BA45 and BA47) and increasingly concrete properties like grasp type being encoded by more posterior sectors (BA44 and BA6) (Kilner, 2011).

References

Baumgaertner, A., Buccino, G., Lange, R., McNamara, A., & Binkofski, F. (2007). Polymodal conceptual processing of human biological actions in the left inferior frontal lobe. *European Journal of Neuroscience, 25,* 881-889. doi:10.1111/j.1460-9568.2007.05346.x

Bornkessel-Schlesewsky, I., & Schlesewsky, M. (2016). The Argument Dependency Model. In G. Hickok & S. Small (Eds.), *Neurobiology of language* (pp. 357-369). New York: Elsevier.

Bornkessel-Schlesewsky, I., & Schlesewsky, M. (2019). Sentence processing: Toward a neurobiological approach. In G.I. de Zubicaray & N.O. Schiller (Eds.), *Oxford handbook of neurolinguistics* (pp. 676-709). New York: Oxford University Press.

Clerget, E., Winderickx, A., Fadiga, L., & Olivier, E. (2009). Role of Broca's area in encoding sequential human actions: A virtual lesion study. *NeuroReport, 20,* 1496-1499. doi: 10.1097/WNR.0b013e3283329be8

Fazio, P., Cantagallo, A., Craighero, L., D'Ausillo, A., Roy, A.C., Pozzo, T., Calzolari, F., Granieri, E., & Fadiga, L. (2009). Encoding of human action in Broca's area. *Brain, 132,* 1980-1988. DOI: 10.1093/brain/awp118

Friederici, A.D. (2016). The neuroanatomical pathway model of language: Syntactic and semantic networks. In G. Hickok & S. Small (Eds.), *Neurobiology of language* (pp. 349-356). New York: Elsevier.

Friederici, A.D. (2017). *Language in our brain: The origins of a uniquely human capacity.* Cambridge, MA: MIT Press.

Hagoort, P. (2014). Nodes and networks in the neural architecture for language: Broca's region and beyond. *Current Opinion in Neurobiology, 28,* 136-141.

Haggort, P. (2016). MUC (Memory, Unification, Control): A model of the neurobiology of language beyond single word processing. In G. Hickok & S. Small (Eds.), *Neurobiology of language* (pp. 339-347). New York: Elsevier.

Kemmerer, D. (2022). *Cognitive neuroscience of language: An introduction.* 2nd edition. New York: Routledge.

Kilner, J.M. (2011). More than one pathway to action understanding. *Trends in Cognitive Sciences,15,* 352-357. doi:10.1016/j.tics.2011.06.005

Matchin, W., & Hickok, G. (2020). The cortical organization of syntax. *Cerebral Cortex, 30,* 1481-1498.

Murphy, E. (2021). *The oscillatory nature of language.* Cambridge, UK: Cambridge University Press.

Pazzaglia, M., Smania, N., Corato, E., & Aglioti, S.M. (2008). Neural underpinnings of gesture discrimination in patients with limb apraxia. *Journal of Neuroscience, 28,* 3030-3041. doi: 10.1523/JNEUROSCI.5748-07.2008

Rizzolatti, G., Cattaneo, L., Fabbri-Destro, M., & Rozzi, S. (2014). Cortical mechanisms underlying the organization of goal-directed actions and mirror neuron-based action understanding. *Physiological Review, 94,* 655-706. doi: 10.1152/physrev.00009.2013
